# Use of effective lids reduces presence of mosquito larvae in household water storage containers in urban and peri-urban Zika risk areas of Guatemala, Honduras, and El Salvador

**DOI:** 10.1186/s13071-021-04668-8

**Published:** 2021-03-19

**Authors:** Jessie Pinchoff, Martha Silva, Kathryn Spielman, Paul Hutchinson

**Affiliations:** 1 Population Council, New York, NY 10017 USA; 2 Tulane University, New Orleans, LA USA; 3 Population Council, Washington, DC USA

**Keywords:** Zika virus, Behavior change, Vector control, Latin America and the Caribbean, Urban, *Aedes aegypti*

## Abstract

**Background:**

In 2015, an outbreak of Zika virus spread across Latin America and the Caribbean (LAC). Public health programs promoted vector control behaviors, including covering water storage containers with lids. Such approaches disrupt Zika transmission by eliminating the habitats of the *Aedes aegypti* mosquito, which breeds in stagnant water.

**Methods:**

A quantitative household survey and observation checklist with trained enumerators were undertaken between August and October 2018 in selected urban/peri-urban USAID implementation communities in El Salvador, Guatemala, and Honduras. The survey included questions regarding knowledge, attitudes, and practices related to Zika virus. An accompanying checklist was implemented to observe water storage containers, including for short-term and long-term water use. The characteristics of these containers were tabulated, including the presence of a lid. The lids were examined for key features to determine their potential effectiveness to prevent mosquito breeding: fully covering and sealing the container, not having holes, and not having water on them (potentially creating a secondary breeding site). Multivariate logistic regression was used to estimate the effectiveness of lid types and characteristics on the presence of larvae.

**Results:**

Overall, in adjusted models, using an effective lid versus no lid was associated with a 94% decrease in odds of larval presence in long-term water storage containers (odds ratio = 0.06; 95% confidence interval [0.029, 0.152]); however, similar impacts were not observed for washbasins in the adjusted models. Models adjusted for household wealth, receiving a visit from a vector control technician, scrubbing the container in the last 7 days, and perception of more mosquitoes around.

**Conclusions:**

Effective lids, if made available and coupled with complementary behavioral messaging, may reduce transmission of Zika and other *Aedes* mosquito-borne diseases in the LAC region. 
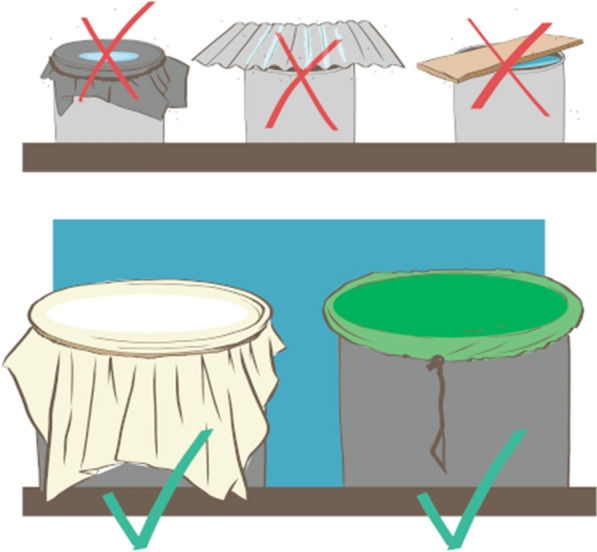

**Supplementary Information:**

The online version contains supplementary material available at 10.1186/s13071-021-04668-8.

## Background

In 2015, the first outbreak of Zika virus was reported in Latin America and the Caribbean (LAC), and was subsequently associated with a spike in congenital malformations (referred to as congenital Zika syndrome) and other neurological complications such as Guillain–Barre syndrome. By August 3, 2017, there were approximately 217,000 confirmed cases of Zika virus in the region [1]. While the outbreak has subsided, Zika is now considered endemic throughout LAC, in addition to parts of Africa and Asia [2]. Although Zika is unique among arboviruses because it can also be transmitted sexually, the virus is mainly transmitted by the *Aedes aegypti* mosquito, the same vector as other arboviruses, including dengue and chikungunya [3]. While several Zika programs in the LAC region direct their programmatic efforts to address both sexual transmission and vector control, the focus for this paper will be household vector control activities.

*Ae. aegypti* mosquitoes are historically a challenge for vector control programs; despite concerted elimination efforts since the 1950s, a resurgence of the arboviral diseases they transmit has been reported in recent years [4, 5]. This resurgence is in large part due to rapid, unmanaged urbanization in tropical cities, human migration, globalization, environmental changes, erratic water supplies leading to water shortages and insecurity, growing insecticide resistance, and ineffective or unsustainable vector control [6–8]. Eliminating *Ae. aegypti* breeding sites by targeting the immature aquatic stages (pupae and larvae) is considered to be one of the most effective household vector control interventions to control transmission of arboviruses such as Zika [7]. However, implementation is challenging, as the *Ae. aegypti* mosquito is highly anthropophilic, can reproduce in small amounts of water (e.g., in a bottle cap), and their eggs can survive being dry for more than a year [6, 9]. With unreliable piped (also known as reticulated) water supply, households in the region store water to fulfill basic needs related to cleaning, cooking, and drinking. This water often reaches the home via a piped water supply system managed by the local government. Although often unsafe for drinking, the piped water should not contain mosquito larvae, yet once stored in containers, the stagnant water may create potential mosquito breeding sites. If source reduction is to be effective, a multidisciplinary response is needed, addressing water access, urban planning, and behavior change strategies at the household and community levels.

In households, there are several behaviors being promoted as part of an integrated vector management strategy. Some require visits from vector control workers (for example, larvicide application), but many can be done by individuals at home [7]. As part of the United States Agency for International Development (USAID) Zika response in the region, social and behavior change programs promote three preventative vector control behaviors: eliminating standing water (e.g., throwing out tires where water accumulates), cleaning water storage containers at least weekly to eliminate eggs from the walls of the container, and covering containers with lids. Studies from other regions have found use of a lids to be effective [10–12]. Container lids are not an absolute barrier, but if tightly fitted, they are shown to be able to prevent gravid female mosquitoes from entering and laying eggs inside the container along the water line [12–14]. While lids can be effective, they require correct application by the user. However, most studies do not detail the characteristics of the lids, only reporting on presence or absence of any lid [15–21]. Additionally, most studies include use of a lid as one component of a multipronged approach, for example in combination with community mobilization efforts [22], school-based information and education campaigns, weekly clearing of stagnant water [23], and larvicide use [24].

Although many *Ae. aegypti* interventions including lids are not new, a recent review states that there is a paucity of reliable evidence regarding what vector control methods are effective for reducing abundance of *Aedes* larvae, particularly with few rigorous study designs such as the randomized trial [6]. Covering water containers with lids has long been promoted to prevent *Ae. aegypti* breeding sites; however, their effectiveness in rigorous research studies is not always clear-cut. For lids to be effective, they should not have holes, should hermetically seal the container, and they should not dip into the water or have water accumulated on them, creating a secondary breeding site on the lid. A study in Thailand found correct use of lids was effective in reducing the presence of larvae in jars used for storing water, defined as the lid fully covering the container [12]. However, the authors found that frequent use of the jars reduced the effectiveness of lids. While frequent emptying of containers can interrupt the mosquito life cycle, removing and replacing the lid too often reduced effectiveness [12]. A separate randomized trial in Mexico and Venezuela assessing the effectiveness of insecticide-treated water container covers found that these significantly reduce the number of larvae detected [16]. Field-based studies often report on the use of water storage container lids as one component of a larger intervention, making it difficult to isolate the effect of the lids. There is also limited description in published studies regarding the characteristics of lids that may make them more (or less) effective.

This is the first study conducted in the LAC context to provide detail on the use of different types of washbasins, containers, and lids and to explore the association between use of an effective lid and the presence of larvae. We explored household use of lids on washbasins and long-term water storage containers (mainly large plastic drums). Data collection involved a household survey and a direct observation checklist tool. Logistic regression modeling was used to estimate the relationships between lids and presence of mosquito larvae, adjusted for household demographic factors and other vector control activities.

## Methods

### Study sites and household survey

**Fig. 1:**
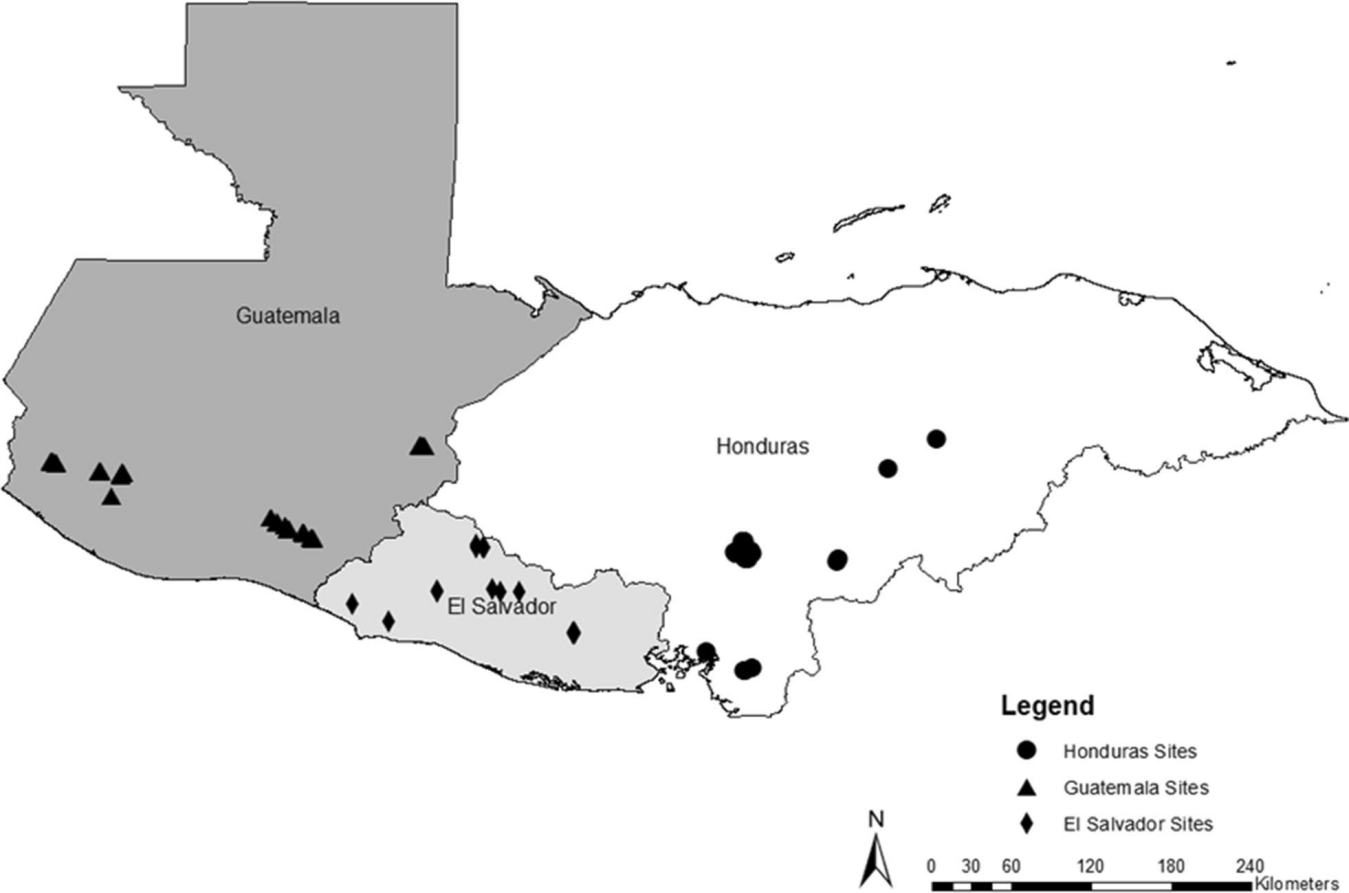
Map of study sites in Guatemala, Honduras, and El Salvador where surveys were conducted

The sampling frame was created from Zika response implementation areas comprised of communities where USAID-funded community engagement partners were working to implement Zika prevention activities in Guatemala, Honduras, and El Salvador (map of sites in Fig. 1). Communities were selected using probability proportional to population size, a multistage sampling methodology where at each stage the probability of selection for each sampling unit is proportional to its size [25]. Electronic maps of selected communities were subdivided into sections using superimposed grids of 500^2^ m. Grid cell centroid points were generated using ArcGIS v10.2, and a randomly selected subset of centroids were used as the starting point for random walks. The number of centroids chosen per community was proportional to the size of the community. The final selection of grid cells to be included in the sample were mapped to get an accurate count of households, which determined the sampling interval per community. Once each starting point was confirmed to be viable, enumerators began a random walk from a selected centroid point, systematically visiting households per the sampling interval until they reached their quota for each centroid. Men and women 18–49 years of age were eligible to participate; in households with multiple eligible adults, enumerators constructed a household roster, and one eligible adult was randomly selected using a random number generator that was built into the electronic survey instrument.

Respondents were invited to participate in an interviewer-administered questionnaire collecting sociodemographic information and knowledge, attitudes, and practices related to Zika, including self-reported household mosquito prevention practices and exposure to health interventions via home visits. Household wealth based on asset ownership was categorized into tertiles using principal components analysis (PCA). Participants provided written informed consent to participate. The survey methods and procedures were reviewed and approved by a local institutional review board (IRB) in each country (the Honduran Institute for Social Security, the El Salvador Ministry of Health, and IRB Zugueme in Guatemala) as well as the Tulane University IRB. The survey was conducted between August and October 2018, the rainy season in this region. In the rainy season, more mosquito larvae are expected to be found at households; thus arboviral diseases such as Zika or Dengue follow a seasonal pattern [1, 26].

### Observation checklist tool

After the household survey, the surveyor inspected all household water storage containers, including washbasins and long-term containers. Washbasins are commonly used throughout LAC; a typical washbasin generally has one or two basins with a washboard and is used daily. Any container used to store water, inside or outside the home, was included in the observation. The checklist included the material of the washbasin or container (e.g., cement, plastic, metal), frequency of use (e.g., every day, less than once per day but more than once per week, less frequently; for washbasins, only), what the stored water was used for (e.g., drinking, cleaning, personal hygiene), and whether the container was covered, followed by observation checklist items about the condition of the lid that were used to create an indicator of “lid effectiveness.” The lid effectiveness indicator included (1) whether the lid had any holes, (2) whether the lid was observed to fully seal onto the container, and (3) whether any water was observed on the lid. If the lid had no observable holes, sealed the container, and had no water accumulated on it, it was considered effective; otherwise, it was not.

Field teams in all three countries were trained to observe water storage containers, including to detect the presence of mosquito larvae. Field teams were trained either by an entomologist from the ministry of health or by a vector control technician employed by a USAID Zika response implementing partner. Field teams were taught to spot larvae using a flashlight to illuminate the inner walls and surface of the water. They recorded the presence or absence of larvae in the observation tool (dichotomous variable) as a simple field-based measure.

### Data analysis

All analyses were implemented using Stata version 15.0. Data were pooled across the three countries. Frequencies were generated to assess household-level characteristics, such as number of containers and washbasins per household or whether the household had received a visit from a vector control worker in the last year. The remainder of the analysis used washbasins and containers as the unit of analysis to assess characteristics including presence of an effective lid. Next, logistic regression models were run to present the associations and magnitude of effects between having an effective, ineffective, or no lid (reference category) on presence of larvae in the washbasin/container, adjusting for cleaning the container in the last 7 days, a set of household-level characteristics, and country. For washbasins, frequency of use (at least once per day vs. two to three times per week vs. less than once per week) was also adjusted for. Models accounted for household-level clustering since some households owned multiple containers or washbasins. Logistic regression models were used due to the binary outcome of presence or absence of larvae. Models were run separately for each independent variable and in combination to ascertain the best model fit and check for any potential interaction effects. Variables included in the fully adjusted model were statistically significant in bivariate models at the *p* < 0.05 level. The models included categorical variables for type of lid, along with a set of control variables hypothesized to also affect the presence of larvae. These include a variable for cleaning of washbasin/container in the last 7 days, frequency of use of washbasin (which could disrupt larval habitats), and perception of mosquitoes around the home. We also included a variable for wealth and country to adjust for unobservable household level factors that might be associated with other mosquito abatement actions, and a variable for visit from a health worker, which might also be associated with knowledge or implementation of promoted behaviors to reduce breeding sites.

## Results

A map of the study sites is depicted in Fig. 1. A total of 1949 men and women were successfully interviewed, with 672 respondents in El Salvador, 609 in Honduras, and 668 in Guatemala. Of these, we conducted observations in 90% of households in El Salvador, 70% in Honduras, and 82% in Guatemala. Ownership of washbasins and containers varied across the countries. Most households owned between one and two containers and one and two washbasins. Less than 5% of households did not own a washbasin (5% in El Salvador, 3% in Honduras, and 2% in Guatemala) (Table 1). There was more variation for containers, with 24% of households in Honduras reporting they did not own a container, as compared with 11% in Guatemala and 4% in El Salvador. The perceived seasonal density of mosquitoes at the time of the survey also varied, with 64% of households in Guatemala reporting a lot of mosquitoes around as compared with 54% in El Salvador and 35% in Honduras (Table 1). Most households had received a visit from someone in the last year (i.e., a technician) to discuss how to prevent mosquito breeding sites in and around the home (59% in El Salvador; 69% in Guatemala; 68% in Honduras). However, there was country-level variation in self-reported preventive behaviors conducted in the last 7 days, with respondents in Guatemala reporting the lowest likelihood of covering or cleaning any washbasin or water storage containers. Only 4% of participants from Guatemala with any type of container reported covering it compared with 45% in El Salvador and 13% in Honduras. Approximately 12% of participants from Guatemala with any type of container said they cleaned it in the last week as compared with 54% in El Salvador and 39% in Honduras (Table 1).

**Table 1:** Characteristics of households surveyed from August to October 2018 from peri-urban locations in El Salvador, Guatemala, and Honduras

	El Salvador	Guatemala	Honduras
	No. (%)	No. (%)	No. (%)
Total households	670	668	609
Avg number of containers/household	1.56 (1.04)	1.75 (1.77)	1.30 (1.66)
Has no containers	4%	11%	24%
Avg number of washbasins/household	1.14 (0.56)	1.10 (0.38)	1.05 (0.37)
Has no washbasins	5%	2%	3%
At this time of year, would you say there are a lot of mosquitoes around (vs. little or no mosquitoes around)	360 (54%)	430 (64%)	210 (35%)
Received a visit from someone talking about vector control in last year	391 (59%)	458 (69%)	404 (68%)
Reports covering household water storage containers/washbasins in last 7 days	298 (45%)	26 (4%)	79 (13%)
Reports scrubbing/cleaning container/washbasins in last 7 days	362 (54%)	83 (12%)	237 (39%)

### Characteristics of household washbasins

**Fig. 2:**
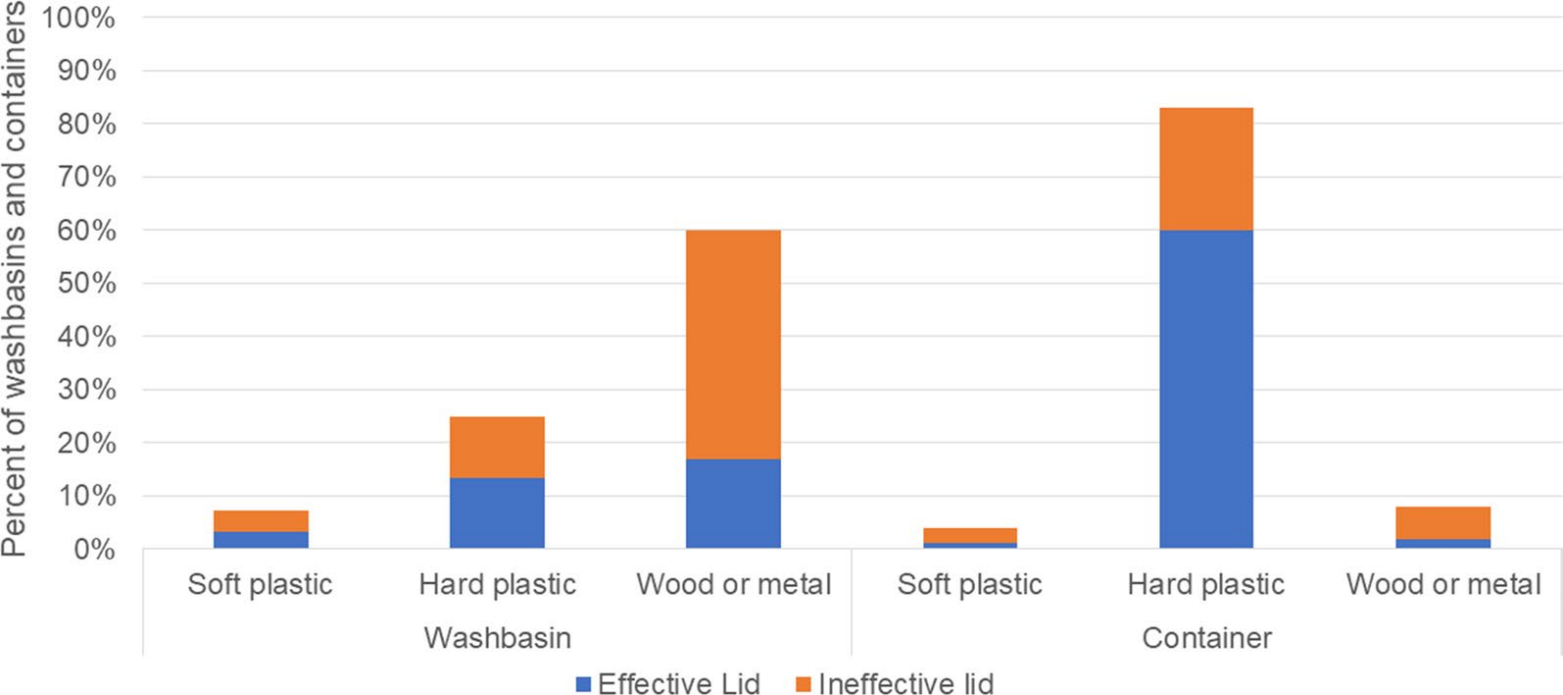
Proportion of washbasin and containers with lids that are effective or ineffective, by material of the lid

**Fig. 3:**
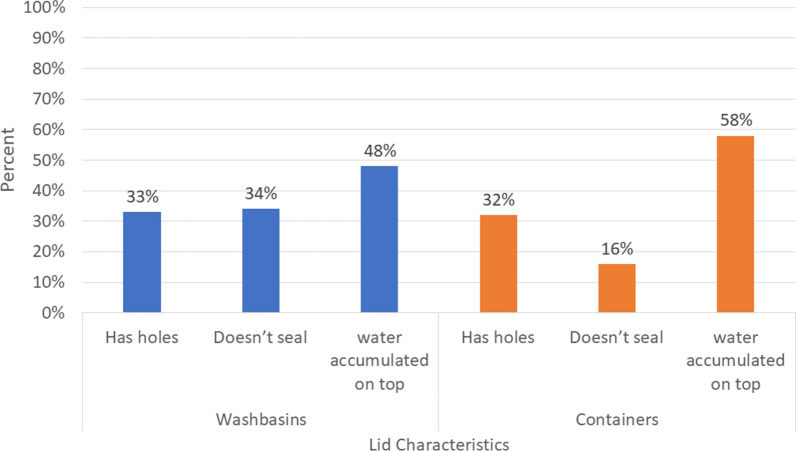
Proportion of washbasin and container lids that are ineffective by three characteristics (has holes, does not seal, water accumulated on top)

Most washbasins, over 90%, were made of cement and were used mainly for household cleaning. Other reported uses included personal hygiene and cooking. Water from washbasins was rarely used for drinking. Most households reported changing the water in their washbasin multiple times per day (versus once per day up to once per week). Not all of the washbasins that were reported to be covered in the interview were actually observed to be covered. Only 1% of washbasins in Guatemala were observed to be covered as compared with 17% in El Salvador and 13% in Honduras (Table 2). Of these, an even smaller proportion were observed to be covered with an effective lid (less than 10% for all three countries). Larvae were observed in 14% of washbasins in El Salvador, 17% in Guatemala, and 13% in Honduras. Overall, washbasins that were covered were most likely to have lids made of wood or metal (60%) (Fig. 2). However, only 44% of wood/metal lids were considered to be effective. The main reasons that washbasin lids were not effective was because they did not fully seal the container (66%), there was water accumulated on the lid creating a potential second breeding site (48%), or the lid had holes in it (33%) (Fig. 3).

**Table 2:** Characteristics of washbasins observed at households surveyed from August to October 2018 from peri-urban locations in El Salvador, Guatemala, and Honduras

	El Salvador	Guatemala	Honduras
	No. (%)	No. (%)	No. (%)
Total washbasins	662	597	480
Washbasin made of cement	600 (91%)	567 (95%)	461 (95%)
What is this water used for?			
Cooking	180 (27%)	27 (5%)	34 (7%)
Drinking	14 (2%)	10 (2%)	4 (1%)
Household cleaning	593 (90%)	575 (96%)	463 (95%)
Personal hygiene	390 (59%)	226 (38%)	348 (72%)
Reports using water in washbasin every day (vs. between once per day and once per week)	621 (94%)	556 (93%)	395 (81%)
Washbasin observed covered	111 (17%)	5 (1%)	62 (13%)
Effective lid observed on washbasin	35 (5%)	3 (1%)	30 (6%)
Larvae or pupae are present	93 (14%)	101 (17%)	65 (13%)

### Characteristics of household containers

Many households reported long-term water storage in a variety of large containers. Most were made of plastic: over 80% in all three countries (Table 3). The water in containers was used for a greater variety of purposes than the water stored in washbasins. The proportion of containers observed with a lid was highest in El Salvador (64%), followed by Honduras (53%) and Guatemala (42%), while the proportions of containers with larvae observed was in the reverse order across countries, with Guatemala the highest (30%), followed by Honduras (18%) and El Salvador (11%) (Table 3). Among washbasins observed to be covered, most lids consisted of wood or metal sheets (60%) (Fig. 2). Among containers observed to have a lid, most were made from hard plastic (83%) (Fig. 2). About 60% of hard plastic lids were considered effective, the highest effectiveness for all lids inspected (Fig. 2). The main reasons that a lid was ineffective were that it did not seal the container (48% of ineffective lids), there was water accumulated on top (19%), or the lid had holes in it (19%) (Fig. 3).

**Table 3:** Characteristics of other household water storage containers observed at households surveyed from August to October 2018 from peri-urban locations in El Salvador, Guatemala, and Honduras

	El Salvador	Guatemala	Honduras
	No. (%)	No. (%)	No. (%)
Containers	332	219	121
Container made out of…			
Cement	22 (7%)	19 (9%)	13 (11%)
Plastic	285 (86%)	178 (81%)	103 (85%)
Metal	25 (8%)	19 (9%)	3 (3%)
What is this water used for?			
Cooking	50 (15%)	7 (3%)	21 (17%)
Drinking	74 (22%)	17 (8%)	0 (0%)
Household cleaning	105 (32%)	101 (46%)	45 (37%)
Personal hygiene	70 (21%)	84 (38%)	47 (39%)
Gardening	18 (5%)	3 (1%)	3 (2%)
The container is observed covered	212 (64%)	91 (42%)	64 (53%)
Effective lid observed on container	142 (43%)	61 (28%)	38 (31%)
Larvae or pupae are present	35 (11%)	63 (30%)	20 (18%)

### Association with mosquito larvae

Table 4 presents the results of logistic regression models, first simple models then fully adjusted models separately for washbasins and containers. The simple models control for scrubbing the container in the last 7 days and the country of the survey to explore the association between use of no lid, ineffective lids, or effective lids on observation of larvae. Fully adjusted models additionally explore receiving a visit from a health worker, reporting higher mosquito abundance, household wealth, and for washbasins, the frequency of use.

**Table 4:** Logistic regression models presenting the association between effective, ineffective lids compared to no lids on presence of larvae among study households sampled in El Salvador, Guatemala, and Honduras from August to October 2018

	Washbasins (*n* = 1735)				Containers (*n* = 654)			
	Model 1^a^		Model 2^a^		Model 3^a^		Model 4^a^	
	OR	95% Confidence interval	OR	95% Confidence interval	OR	95% Confidence interval	OR	95% Confidence interval
No lid	Ref.		Ref.		Ref.		Ref.	
Ineffective lid	1.222	0.714, 2.091	1.174	0.672, 2.053	0.388**	0.214, 0.703	0.410***	0.223, 0.752
Effective lid	0.36*	0.128, 1.000	0.336	0.109, 1.031	0.067***	0.028, 0.156	0.064***	0.029, 0.152
Received a visit from a health worker	–	–	0.542***	0.403, 0.728	–	–	0.455**	0.271, 0.764
Wealth tertile 1, (poorest)	–	–	Ref.		–	–	Ref.	
Tertile 2 (medium)	–	–	0.805	0.570, 1.137	–	–	1.452	0.819, 2.577
Tertile 3 (wealthiest)	–	–	0.587**	0.397, 0.867	–	–	1.094	0.536, 2.234
More mosquitoes around (vs. same or fewer)	–	–	1.705**	1.240, 2.346	–	–	1.350	0.792, 2.301
Scrubbed container in last 7 days	0.322***	0.229, 0.452	0.378**	0.256, 0.557	0.439**	0.258, 0.746	0.483*	0.260, 0.899
Frequency of use (every day, vs. less frequent use)	–	–	2.309***	1.503, 3.547	NA		NA	

NA this variable was not available for analysis

^a^All models adjust for country

Statistical significance denoted as *p < 0.05; **p < 0.01; ***p < 0.001

For washbasins, using an effective lid was associated with 64% lower odds of larvae (OR = 0.36; 95% confidence interval [0.128, 1.00]) in the simple model; however, this effect was not significant in the fully adjusted model (Table 4). In fully adjusted models, households that received visits from a health worker and were in the highest wealth tertile were less likely to have larvae observed in their washbasins. Households were more likely to have larvae in the washbasin if they reported more mosquitoes around [OR = 1.705; 95% CI (1.240, 2.346)]. Washbasins were more likely to have larvae if they were used infrequently compared to every day [OR = 2.309; 95% CI (1.503, 3.547)].

For containers, the odds of larval presence were significantly lower when using an effective lid in both simple and adjusted models. In the adjusted models, using an effective lid was associated with 93% lower odds of larvae [OR = 0.064; 95% CI (0.029, 0.152)] (Table 4). Using an ineffective lid was associated with 59% lower odds of larvae [OR = 0.410; 95% CI (0.029, 0.152)]. While receiving a visit from a health worker was associated with lower odds of larval presence in containers, wealth and reporting more mosquitoes around were not. Cleaning the container in the last 7 days reduced the odds of larvae being observed in both washbasins and containers in fully adjusted models. Models were run adjusting for larvicide application but were not significantly different  (Additional file 1: Table S1).

## Discussion

Washbasins and other containers for storing water in or near homes in urban and peri-urban LAC are ubiquitous, creating ideal breeding sites for the *Ae. aegypti* mosquito and potentially contributing to the transmission of arboviruses such as Zika. Social and behavior change campaigns commonly target two household-level behaviors targeting these washbasins or containers: cleaning water containers weekly and covering water containers to reduce Zika transmission. Our findings suggest that use of an effective lid on long-term water storage containers is associated with reduced odds of mosquito larval presence, even after adjusting for cleaning water containers.

To date, there is very little evidence regarding the effectiveness of lids in the LAC region and in the context of Zika virus transmission. Almost no available research describes the characteristics of lids themselves. Our study includes details of the characteristics of the lids and finds a statistically significant lower odds of larval presence, particularly in long-term storage containers that use an effective lid. A recent systematic review and meta-analysis found a very limited number of studies exploring the efficacy of lids in preventing arbovirus transmission (due to the recent introduction of Zika to the LAC region, most of the published literature is focused on other arboviruses such as dengue, but should relate to Zika since *Ae. aegypti* is the same vector), but they suggest environmental management combined with covering water containers is needed to reduce the risk of transmission [6]. Other studies have demonstrated that covering containers and water tanks is effective [14, 24].

The mixed findings in the literature may in part be due to a lack of specificity regarding the type of water storage container and the characteristics of the lids being used. None of the studies reviewed provided details on the lid characteristics, just presence or absence of a lid, an important limitation that our study addresses. Also, few, if any other research studies isolated the effects of solely using a lid to prevent breeding sites; most included covering water containers as one component of a larger community-based prevention campaign. Our analysis highlights the use of lids as an independent vector control strategy.

Overall, we found a very small proportion of the washbasins were covered, likely because they are very difficult to cover due to their shape. In addition, the water in washbasins is used frequently, daily in most cases, so these are perhaps considered lower risk. Our findings support this, as washbasins used infrequently had over twice the odds of having mosquito larvae, compared to washbasins used daily. However, we still found larvae in washbasins (14% in El Salvador, 13% in Honduras, and 17% in Guatemala), suggesting that even though the water was frequently disturbed, these can still be mosquito breeding sites. The main reason we found washbasin lids to be ineffective was because they had water accumulated on them, creating potential secondary breeding sites. New lids designed specifically for washbasins may be more effective if they seal containers completely and do not allow water to accumulate on top of them.

Larvae were more commonly observed in water storage containers than in washbasins. This likely reflects two countervailing forces; while water containers are more easily effectively covered to prevent the vector from entering to lay eggs, they are also more likely to go undisturbed for longer periods, allowing larvae to emerge if eggs were laid. Between 28% (in Guatemala) and 43% (in El Salvador) of covered containers had an effective lid, the great majority of which were made of hard plastic. Containers examined in this study were considered long-term water storage containers.

There are several potential reasons for the low use of lids and the low proportion of these lids observed to be effective: lack of access to or availability of lids, perhaps related to the costs of lids themselves; low perceived risk of mosquito-borne diseases; lack of information (regarding water storage containers as possible breeding sites, how to identify high-risk containers and/or how to prevent breeding sites); or a lack of acceptability of available lids. Lack of acceptability has been documented in trials using insecticide-treated covers. One study in Venezuela tested uptake of insecticide-treated container covers and found that only 21.5% of households accepted the covers, and only 10% were still using them at 22 months, with the main reason for discontinued use being that they had become dirty or damaged [19]. Additional research regarding the availability, pricing, quality, and acceptability of lids should be explored to inform behavior change messaging and campaigns. Additional resources and infrastructural interventions may be required to address water security and sanitation concerns in the region, as these are key drivers of unsafe water storage practices [8, 27].

There are several limitations in this study. First, the sample size per country is relatively small. Second, while most households allowed observation of their water storage containers and washbasins, refusals may have resulted in selection bias. Third, while the field team did receive training on observation of larvae, they themselves were not entomologists, potentially resulting in nonrandom error if earlier containers with low numbers of larvae were missed and reported as having none. Relatedly, we only captured a binary variable for larval presence, which missed any significant variation in larval density. In addition, we did not ask about the origin of the water found in washbasins and containers, which limits our ability to detect variation in likelihood of larval presence based on water source. Despite this, the most common source of water was from a piped water source (over 75%) that would not contain mosquito larvae. Not collecting more information (specifically, the mosquito species) also limited our conclusions regarding disease transmission; however, our main goal was to describe vector control behaviors undertaken by households. Fourth, while the lid and larvae were observed, other variables were self-reported by the interviewed household member and may not reflect the perceptions or recall of all household members. Relatedly, while we did run the models adjusting for larvicide use, we did not present these models because a) not all household members may know if this was done and b) larvicide is a general term comprising multiple products, some effective and some very ineffective, making interpretation of results challenging. Fifth, the cross-sectional nature of these observations does not allow for measurement of consistency. Our findings suggest an ineffective lid may reduce the odds of mosquito larvae found, and effective lids have an even more significant effect; however, this should be interpreted with caution. For example, one study suggests that ineffective lids may be associated with *more* presence of mosquito larvae not less [12].

## Conclusions

In conclusion, our study documents several key characteristics of the lids used to cover washbasins and containers in urban and peri-urban settings in LAC after the Zika outbreak. Despite significant promotion in the region to use a lid and clean containers, lid use was low, and particularly low for washbasins. Our findings suggest using an effective lid significantly reduces the odds of mosquito larvae being present, after adjusting for reported cleaning of the container and household characteristics. The implications of this study are important as arbovirus transmission is increasing in the region and lids are commonly promoted. Behavior change campaigns should integrate messages about specific lid characteristics that make them effective to ensure their effective use. Promoting the use of effective lids for vector control may contribute to reducing Zika transmission in the region.

## Supplementary Information

**Additional file 1: Table S1.** Adjusted logistic regression models presenting the association between effective, ineffective lids compared to no lids on presence of larvae (adjusting for self-reported use of larvicide) among study households sampled in El Salvador, Guatemala, and Honduras from August to October 2018.

## Data Availability

The data set generated during the current study will be available on the USAID Development Experience Clearinghouse (DEC).
