# Supplementary material for: Use of effective lids reduces presence of mosquito larvae in household water storage containers in urban and peri-urban Zika risk areas of Guatemala, Honduras, and El Salvador
Source: Parasit Vectors. 2021 Mar 19;14:167. doi: 10.1186/s13071-021-04668-8 (PMC7977570; doi:10.1186/s13071-021-04668-8)
Supplement: Supplementary file 1 — Additional file 1: Table S1. Adjusted logistic regression models presenting the association between effective, ineffective lids compared to no lids on presence of larvae (adjusting for self-reported use of larvicide) among study households sampled in El Salvador, Guatemala, and Honduras from August to October 2018. [file 13071_2021_4668_MOESM1_ESM.docx]

Supplemental Table 1: Adjusted logistic regression models presenting the association between effective, ineffective lids compared to no lids on presence of larvae (adjusting for self-reported use of larvicide) among study households sampled in El Salvador, Guatemala and Honduras from August to October 2018

|  | Washbasins | |  | Containers | |
| --- | --- | --- | --- | --- | --- |
|  | OR ^a^ | 95% CI |  | OR ^a^ | 95% CI |
| No lid | REF |  |  | REF |  |
| Ineffective Lid | 1.167 | .668, 2.04 |  | 0.432** | 0.216, 0.866 |
| Effective Lid | 0.337 | .110, 1.04 |  | 0.047*** | 0.014, 0.158 |
| Received a visit from a health worker | 0.542*** | .403, 0.728 |  | 0.526* | 0.277, 0.996 |
| Wealth Tertile 1, (poorest) | REF |  |  | REF |  |
| Tertile 2 (medium) | 0.805 | .570, 1.137 |  | 1.319 | 0.645, 2.696 |
| Tertile 3 (wealthiest) | 0.587** | .397, 0.867 |  | 1.132 | 0.493, 2.599 |
| More mosquitoes around (vs same or fewer) | 1.704** | 1.239, 2.345 |  | 0.988 | 0.524, 1.864 |
| Scrubbed container in last 7 days | 0.378*** | .256, 0.557 |  | 0.519 | 0.250, 1.075 |
| Used larvicide in last 7 days | 1.017 | .543, 1.907 |  | 1.142 | 0.479, 2.724 |
| Frequency of use (every day, vs less frequent use) | 2.315*** | 1.507, 3.556 |  | NA |  |

^NA^ this variable was not available for analysis

^a^ All models adjust for country

Statistical significance denoted as * p<0.05; ** p<0.01; *** p<0.001
